# Financial Pressures of Family Caregiving

**DOI:** 10.1093/geroni/igaf122.1187

**Published:** 2025-12-31

**Authors:** Selena Caldera

**Affiliations:** AARP, Washington, District of Columbia, United States

## Abstract

As in the 2020 Caregiving in the US survey, one in five family caregivers experienced high financial strain due to their caregiving responsibilities. This strain may result from out-of-pocket care costs, reduced work hours, or a combination of factors. The 2025 Caregiving in the US report provides a detailed look at the financial impacts that family caregivers experience. Caregivers report a range of financial impacts, including debt, savings, and housing-related issues. Lower-income family caregivers (those with less than $50,000 in household income) are significantly more likely to experience all thirteen financial impacts identified in the survey. Younger caregivers also face more financial challenges due to caregiving. Both groups often start their caregiving journey with fewer savings and assets, making them more vulnerable to long-term financial insecurity. This presentation will summarize the financial strain and impact measures reported by caregivers in 2025 and highlight the groups most likely to experience financial pressures. Caregivers overwhelmingly support policy solutions such as caregiver income tax credits, paid family leave, and programs to pay family caregivers for their care. Understanding both the overall financial pressures and the caregivers hardest hit is critical to developing effective policy solutions to support all caregivers financially.

